# Healthy Eating for All? The Challenge of Adhering to Dietary Guidelines for Low-Income Groups in China

**DOI:** 10.3390/nu15122704

**Published:** 2023-06-09

**Authors:** Jingjing Yin, Jingfen Hua, Xinhuan Zhang, Alexandre Tuyishimire, Degang Yang

**Affiliations:** 1 Key Lab of Urban Environment and Health, Fujian Key Laboratory of Watershed Ecology, Institute of Urban Environment, Chinese Academy of Sciences, Xiamen 361021, China; jjyin@iue.ac.cn (J.Y.); jfhua@iue.ac.cn (J.H.); alexandre@iue.ac.cn (A.T.); 2 State Key Laboratory of Desert and Oasis Ecology, Key Laboratory of Ecological Safety and Sustainable Development in Arid Lands, Xinjiang Institute of Ecology and Geography, Chinese Academy of Sciences, Urumqi 830011, China; dgyang@ms.xjb.ac.cn; 3 University of Chinese Academy of Sciences, Beijing 101408, China

**Keywords:** food prices, affordability, calorie-adequate, balanced diet, low-income group

## Abstract

The Chinese Dietary Guidelines propose a balanced diet for healthy living, but the affordability of this diet needs to be considered, especially for low-income households. To investigate the affordability of a healthy diet, this study analyzes the daily retail prices of 46 food items in 36 Chinese cities from 2016 to 2021. This study compares expenditure, diet composition, and nutritional status in two scenarios aligned with the guidelines. The results show that the mean minimum cost of a balanced diet exceeds the current per capita food expenditure for at least 182.85 million urban households. This suggests that low-income people would need to increase their expenditure by at least 20–121% to achieve the recommended diets. This study also identifies affordable and nutrient-dense foods such as standard flour, eggs, black beans, and cabbage, which policymakers should focus on when monitoring food prices. The findings recommend a combination of social and food system policies to reduce prices and make healthy diets accessible. This study identifies the gaps in the Chinese Dietary Guidelines for ensuring accessibility for vulnerable groups and provides a template for policymakers and researchers to track diet affordability using available food price data in China, contributing to China’s 2030 Health Plan and the UN’s Sustainable Development Goals.

## 1. Introduction

Food security, as defined by the FAO, is “physical and economic access for all people at all times to sufficient, safe and nutritious food to meet their dietary needs and food preferences for an active and healthy life” [1]. Affordability, which is an important attribute of food security, is a key barrier to accessing adequate, safe, and nutritious food for active and healthy living [2], and it is particularly evident in poor countries and among poor groups. In India, the cost of a healthy diet exceeds the international poverty line (USD 1.90 per person per day in purchasing power parity (PPP) terms), making it unaffordable for the poor [3]. In South Asia, approximately 38% of the population cannot afford to eat healthily [4]. In Africa, there are 149 million people who cannot afford to meet their basic energy needs for a proper diet [2]. The COVID-19 outbreak in 2020 exacerbated global food insecurity, with preliminary assessments suggesting that the COVID-19 outbreak could increase the total number of undernourished people globally from 83 million to 132 million in 2020 [5]. A potentially significant cause of this impact was the shock to food production activities and supply chains during the COVID-19 outbreak, which led to a spike in the food prices [6], thereby reducing the demand for nutritious food, as the largest increases were in vegetables and animal foods, which are the main sources of essential micronutrients in the diet [6,7,8,9]. The outbreak of COVID-19 therefore exacerbated the shift toward monotonous, malnourished diets among consumers, especially vulnerable groups [8]. The two most recent editions of the United Nations’ annual report of the State of Food Security and Nutrition in the World adopted the cost of a healthy diet as an indicator to assess food security, concluding in SOFI 2021 that more than three billion people worldwide cannot afford a healthy diet [5].

In general, a healthy diet that is nutritionally adequate is more costly than an energy-sufficient diet [10,11,12,13]. According to FAO data, the average global cost of a healthy diet is more than four times the cost of the most affordable starchy staple food required to meet daily energy needs [2]. Food prices influence consumption choices, and therefore, overall diet quality and nutrition, leading to stunting and obesity [14,15]. It is certain that the nutritional health of the poor is more likely to be affected by food prices [16], as the amount of food that poor households can afford is constrained by their low incomes. Studies confirm that poor individuals have less dietary diversity than wealthier individuals [11,17], and people in poverty rely more on relatively inexpensive starchy staples and less on vegetables and animal foods [17,18]. However, studies also concluded that the poor in some areas rely more on healthy vegetables and pulses than the middle class [19].

Existing studies have generally clarified the affordability of healthy diets by assessing the lowest costs of calorie-sufficient diets [10,12], diverse diets [12], or nutritionally adequate diets [15,20]. However, to our knowledge, few studies assessed the affordability of nutritious diets for Chinese households. “Hidden hunger” in the Chinese population is becoming a public health concern, with the intake of zinc, iron, vitamin A, thiamine, and riboflavin remaining far below the recommended values [21]. Over 50% of Chinese adults in 2015 still had retinol equivalent, thiamin, and vitamin C intakes below the estimated average requirements, and the proportion of adults with inadequate riboflavin and calcium intakes exceeded 85% and 95%, respectively [22]. To improve the nutritional status of the Chinese population, the Chinese Nutrition Society has published four editions of the Chinese Dietary Guidelines (CDG) since 1989. The 2022 edition proposes a balanced diet that can best meet the nutritional and health needs of healthy people of different ages and energy levels [23]. However, the affordability of diets that meet nutritional needs for low-income groups needs to be further explored.

This study fills this gap by estimating the minimum cost required to achieve a CDG-based healthy diet and comparing it with the actual food expenditure of different income groups to explore the affordability of a healthy diet for the population. We consider the dietary costs of meeting basic caloric needs and meeting food diversity needs as the lower and upper limits of a healthy diet, respectively. We then compare their dietary cost, composition, and nutrient levels to provide practical dietary choices for vulnerable groups. This paper is organized as follows: Section 2 describes the data and methodology used in this study. Section 3 presents a comparison of the cost, diet composition, and nutritional status in different scenarios in line with the CDG. Section 4 provides a discussion of the results and offers relevant policy recommendations. Section 5 presents the concluding remarks.

## 2. Materials and Methods

### 2.1. Data Source

The retail food price data in this study were obtained from the Wind database (https://www.wind.com.cn/ (accessed on 12 September 2022)), which collects monthly average retail food prices from 36 large- and medium-sized cities in China (including 27 provincial capitals, 4 municipalities, and 5 cities with independent planning). A total of 46 food items were included in the dataset, including 10 starchy staples (including 5 whole grains), 3 legumes, 4 oils and fats, 6 meats, 2 fish and seafood, 3 fruits, 16 vegetables (including 5 dark vegetables), eggs, and milk. This study covers the period from 2016 to 2021 and relies on evaluating the difference between actual annual per capita food expenditures among different income groups in urban areas and the least cost calculated in this study to assess food affordability. Our emphasis on the lowest-income and low-income populations in our study is based on their vulnerability to food insecurity, and the data on urban household consumption expenditure by income bracket are calculated based on the China Urban Life and Price Yearbook [24]. To eliminate any potential biases resulting from changing price levels over the study period, we deflated historical food expenditure data using urban Consumer Price Indices (CPIs) and converted annual expenditure data to monthly expenditure levels. Nutritional composition data for each item and the edible portion used in this study were derived from the China Food Composition Tables [25,26]. A detailed list of each food item’s nutritional composition is presented in Supplemental Table S1. Unless otherwise stated, the currency unit used in this paper is the Chinese yuan (CNY).

### 2.2. Methods

#### 2.2.1. Calculation of the Minimum Cost Diet

The dominant method for calculating the minimum cost of diets is usually linear programming, which is based on the concept of least-cost diets developed by Stigler [27], and has long been used by researchers to find the least-cost combination of foods to satisfy the constraints [10,12]. The disadvantage of this method, however, is that it can lead to a large deviation from the actual eating pattern and a lack of practical significance [3]. Therefore, to ensure that the proposed dietary pattern can meet both basic nutritional requirements and households’ dietary habits, the balanced diet based on the CDG is used as a benchmark to set constraints. This approach also contributes to policy coherence, as it shows whether dietary advice based on expert opinion and government decision making is affordable for households. The balanced dietary pattern, which serves as the core of the CDG, specifies the quantities of each food group at energy levels ranging from 1000–3000 kcal, aiming to meet the nutritional and health needs of healthy individuals with varying energy needs. According to the latest China Health and Nutrition Survey (CHNS) conducted in 2018, the daily energy intake of urban male and female residents was 2231.0 ± 747.5 kcal (male) and 1826.4 ± 581.5 kcal (female) [28]. Therefore, this study simulates the following two diet scenarios with the lowest cost at a 2000 kcal energy intake level: the scenario that meets the requirements of a balanced diet (S_BD_), and the scenario of the calorie-adequate diet (S_CA_). These two scenarios, respectively, represent a balanced diet for health (upper bound) and a basic diet for subsistence (lower bound). Our goal is to reveal the trade-off between nutritional adequacy and affordability faced by low-income groups by analyzing the costs, nutritive values, and dietary diversity of different dietary scenarios. To avoid the S_CA_ consisting only of the cheapest cereals, this study constrained five essential food groups in accordance with the CDG [23]. These groups were starchy staples (SS); vegetables and fruits (V&F); meat, fish, and eggs (MFE); legumes and dairy (L&D); and oils and fats (O&F). The food group settings for S_CA_ and S_BD_ correspond to the food groups and food subgroups in Table 1, respectively.

The calculations for cost of the calorie-adequate diet (CoCA) and cost of the balanced diet (CoBD) are based on the technological assistance tools developed by Tufts University [29]. The specific steps are as follows: (a) select the CDG as a reference for calculation; (b) classify foods of the same type into one food group based on retail price data collected, and convert food prices from unit weight to unit energy of the edible portion; (c) on the basis of the restriction for calorie or food diversity, calculate the recommended amount to purchase per day for each food; (d) calculate the cost per day for the purchase of this quantity of each food item at retail prices; (e) select the least expensive foods in each food group on the basis of the cost; (f) sum up the costs of all food groups for the calculation of S_CA_ and S_BD_. All calculations are performed in Stata 16.0.

#### 2.2.2. Affordability of Diets

Diet affordability was measured by “least cost/income” in previous studies [4,10,20,30]. While Chinese people spend much of their disposable income on savings and non-food necessities, such as housing, education, and healthcare, with limited income, food expenditure may be prioritized for reduction, and dietary nutrition levels may decline. Therefore, the use of data on real expenditure on food is a more realistic measure of the affordability of meals than disposable income. The specific formulas are as follows:

(1) $$Aff_{S_{CA},t}=CoCA_{t}/FC_{i,t}$$

(2) $$Aff_{S_{BD},t}=CoBD_{t}/FC_{i,t}$$

Here, $Aff_{S_{CA},t}\;$ and $Aff_{S_{BD},t}$ denote the affordability of *S_CA_* and *S_BD_* in *t* period, respectively. $FC_{i,t}$ is the food cost of *i* income group in t period, and *i* includes the lowest-income and low-income groups. If $Aff_{S_{CA},t}$ (or $Aff_{S_{BD},t}$) > 1, it means that the shift to a calorie-adequate diet (balanced diet) to meet dietary recommendations for the vulnerable groups will increase food expenditure, i.e., the *S_CA_* (*S_BD_*) diet is less affordable and potentially threatens food security. Conversely, it means that the diet is affordable at the current expenditure.

#### 2.2.3. Assessment of Dietary Nutrition Quality

Healthy Eating Index (HEI). The HEI is one of several international indicators used to measure dietary health. Along with the Diet Quality Index (DQI) and Mediterranean Diet Quality Index (MDQI), the HEI is widely used to assess individuals’ diets [31]. It is a set of scores that compares recommended and restricted food groups to actual intake. The HEI draws on analytical frameworks from existing studies [31,32] and has 14 main indicators (listed in Supplemental Table S3) based on the balanced dietary composition of the CDG. We assigned weightings to the indicators based on their nutritional contribution and multiplied them by the standard portion (SP) of each food group to calculate the HEI score. Details about the weighting and SP calculation can be found in the Supplementary Materials.

Mean Adequacy Ratio (MAR) and Mean Excess Ratio (MER). To conduct a comprehensive assessment of dietary nutrient levels, in addition to the evaluation method based on food type, a nutrition-based assessment method was also adopted in this study. The key aim of this method is to calculate the MAR of beneficial nutrients and the MER of restrictive nutrients [33,34]. The daily dietary intake levels of energy, macronutrients, and micronutrients of households were first calculated with reference to the Chinese Food Composition Table [25,26]. The formula is as follows:

(3) $$N_{i}=a_{11}x_{1}+a_{12}x_{2}+a_{13}x_{3}+\ldots +a_{ij}x_{j}$$

where $N_{i}\;$ denotes the daily intake of nutrient *i*, $x_{j}$ denotes the daily intake of food item *j*, and $\;a_{ij}$ represents the amount of nutrient *i* in per unit weight of food item *j*.

To calculate the MAR, we evaluated and added up the differences between the actual intake of each nutrient and the reference standard, using the Recommended Nutrient Intake (RNI) of 14 important and beneficial nutrients as our guide (Supplemental Table S4). The formula is as follows:

(4) $$MAR=\left(\frac{1}{14}\overset{14}{\underset{n=1}{\sum }}\frac{N_{bn}}{RNI_{bn}}\times 100\right)$$

where $N_{bn}$ denotes the daily intake of each beneficial nutrient and $RNI_{bn}$ is the corresponding recommended quantity for this nutrient. The value is set to 1 when $N_{bn}/RNI_{bn}>1$, so that a high intake of one nutrient cannot compensate for a low intake of another, thus affecting the accuracy of nutrition evaluation [34].

The tolerable upper intake (UL) level for three nutrients, namely, fat, saturated fatty acids, and sodium, were used as reference standards to calculate the MER (Supplemental Table S4). Cholesterol is not included here because the upper limit of cholesterol was removed from the Chinese Dietary Nutrients Reference Intakes (2013 Edition) [35], as studies show no correlation between cholesterol intake and the risk of stroke, coronary heart disease, and death [23]. MER is calculated based on the following formula:

(5) $$MER=\left(\frac{1}{3}\overset{3}{\underset{n=1}{\sum }}\frac{UL_{rn}}{Q_{rn}}\times 100\right)-100$$

where $Q_{rn}$ is the intake of restricted nutrients, and $UL_{rn}$ is the corresponding recommended maximum intake value. In the same logic as the MAR calculation, the value is set to 1 when $UL_{rn}/Q_{rn}>1$, to ensure that high intakes of a nutrient are not offset by other nutrients.

## 3. Results

### 3.1. Dietary Affordability Assessment

**Figure 1:**
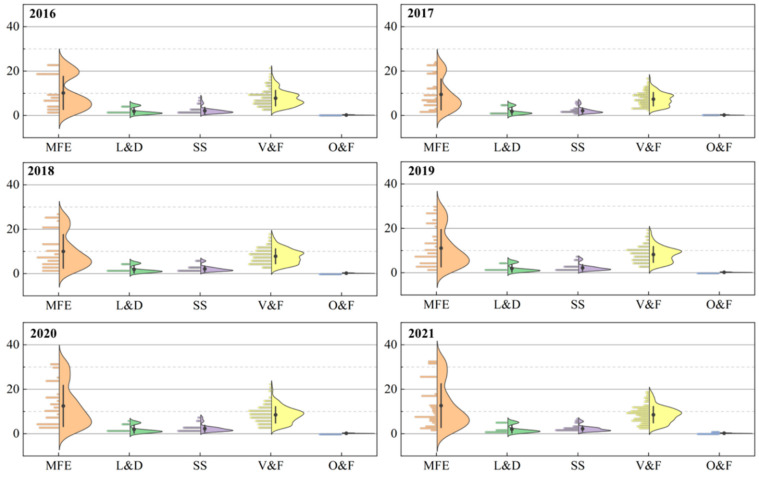
Food costs per balanced diet by five food groups from 2016 to 2021 (CNY). The data were calculated based on the calorie intake level of each food group multiplied by the unit calorie price of each food group under a balanced diet. The graphs show the distribution of the data and the probability densities. The dotted line shows the mean (solid dots) and standard deviation (line).

Figure 1 shows the distribution of the cost across the five food groups that make up a balanced diet. The results confirm that the cost is higher for MFE and V&F, and lower for SS and O&F. In terms of the cost variation, the average cost of MFE was higher during the study period, with costs ranging from CNY 1.46 for bone-in pork to CNY 32.51 for bone-in mutton. The large cost variation leads to a double-crested violin shape in the data point distribution. The cost distribution of both L&D and SS also shows a double-peaked violin, mainly because L&D includes milk and legumes, and the cost of consuming the equal calories from milk is 4.8 times more than that from legumes (Supplemental Table S2), so milk and legumes form the upper and lower peaks, respectively; similarly, SS includes cereals and potatoes, and the cost of the same amount of calories from potatoes is 2.7 times more than that from cereals, so the two foods together form the double peak. The average cost of V&F is similar to that of MFE, but is relatively concentrated, mainly in the range from CNY 2.17 for garlic to CNY 22.23 for tomato, with the average price increasing by 10.2% between 2016 and 2021.The price volatility in SS and O&L is relatively stable, which is mainly due to the strict monitoring and regulation of grain and edible oil prices in China.

**Figure 2:**
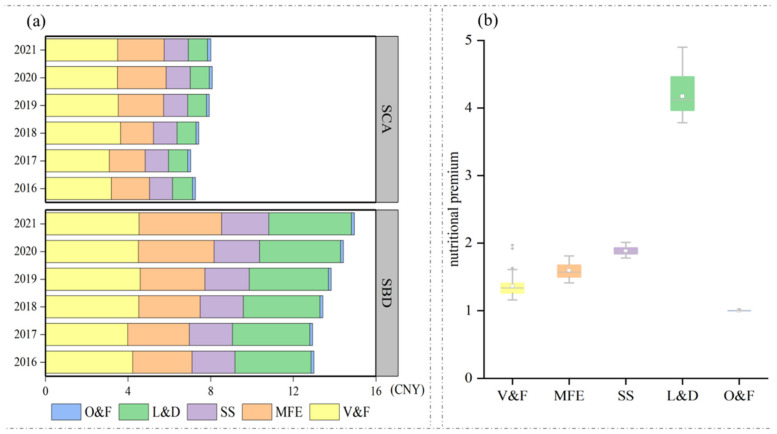
Cost by food group in scenario of the calorie-adequate diet (S_CA_) and balanced diet (S_BD_) (**a**), and the statistical distribution of the nutritional premium (**b**).

Figure 2a reports the total costs of the S_CA_ and S_BD_ and the components by food group. The average daily diet cost during 2016–2021 in the S_CA_ is CNY 7.63 (IQR 7.20–8.02); the highest cost is in 2020 (CNY 8.14, IQR 7.95–8.18); and the highest cost is 14.7% higher than the lowest cost in 2017 (CNY 7.04, IQR 6.98–7.14). The average daily diet cost during 2016–2021 in the S_BD_ is CNY 13.78 (IQR 12.97–14.50); the highest cost is in 2020 (CNY 14.73, IQR 14.45–14.88); and the highest cost is 15.2% higher than the lowest cost in 2017 (CNY 12.79, IQR 12.72–12.84). It can be concluded that the COVID-19 outbreak in 2020 caused a brief spike in food prices, thereby increasing the average daily cost of food. In both the S_CA_ and S_BD_, the food group with the largest share cost is V&F, while the food group with the smallest share is O&F. MFE and L&D are the second largest food groups in the S_CA_ and S_BD_, respectively, and the shares of SS expenditure are similar and stable in both the S_CA_ and S_BD_, reflecting the low price elasticity of SS.

We also explored the proportional premium for dietary health over basic energy (Figure 2b), which is expressed as the ratio of the cost of S_BD_ to S_CA_. The average premium value for 2016–2021 is CNY 1.81, ranging between CNY 1.68 and 1.95. Across food groups, we see a considerable variation in the premium for nutrition, with the highest premium observed in L&D (IQR 3.81–4.38). The premium comes mainly from dairy, as the S_BD_ includes both legumes and milk to meet the food diversity requirement in the balanced diet, while the S_CA_ only includes legumes to limit costs. Across time periods, the nutrition premium was the highest in 2020 and 2021, suggesting that the pandemic may have caused residents to reduce the nutrition of their diets under cost constraints.

**Figure 3:**
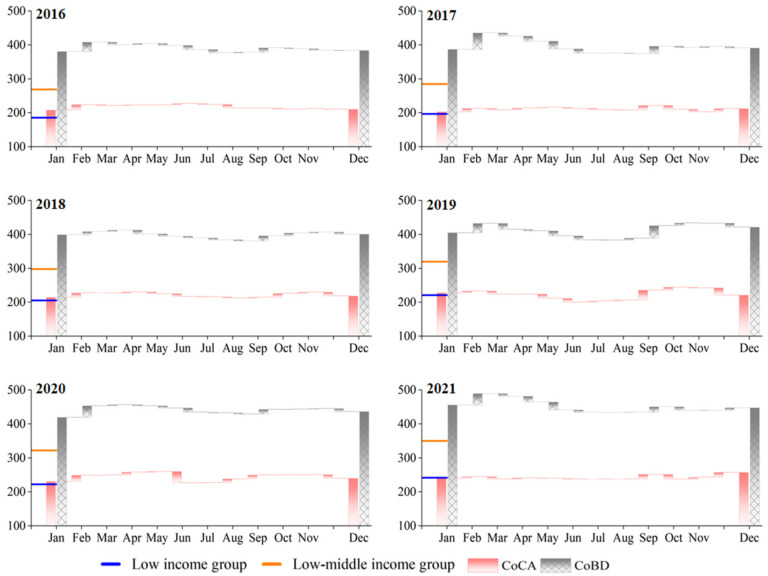
Changes in minimum dietary costs in S_CA_ and S_BD_ and comparison with actual per capita food expenditure for the lowest-income and low-income groups (CNY).

This study calculates the average monthly food expenditure (excluding alcohol and beverages as well as the cost of food processing services and dining out) for the lowest-income and low-income groups and compares it with the monthly cost in the S_CA_ and S_BD_ to reflect the gap between the expenditure under current dietary patterns and the expenditure to meet basic calorie and food diversity requirements. Figure 3 shows that the food expenditure of the lowest-income group accounted for 93.7% (IQR 89.1–97.8%) and 51.2% (49.4–53.0%) of the CoCA and CoBD, respectively, which means that the average daily dietary energy intake and the energy distribution structure of the lowest-income group did not meet the requirements of a balanced diet and were far below the requirements for a balanced diet. The food expenditure levels of the low-income group were 135.7% (129.1–141.7%) of the CoCA and 74.1% (71.6–76.8%) of the CoBD, respectively, indicating that although the low-income group can afford a basic diet with sufficient energy intake based on their current actual spending on food, they cannot meet the requirements for a nutritionally rich and diverse diet as stipulated by balanced dietary patterns. Overall, the lowest-income and low-income households would need to increase their dietary expenditure by 20–121% above their current spending levels to achieve a healthy and balanced diet recommended by the CDG.

### 3.2. Dietary Composition Change

**Figure 4:**
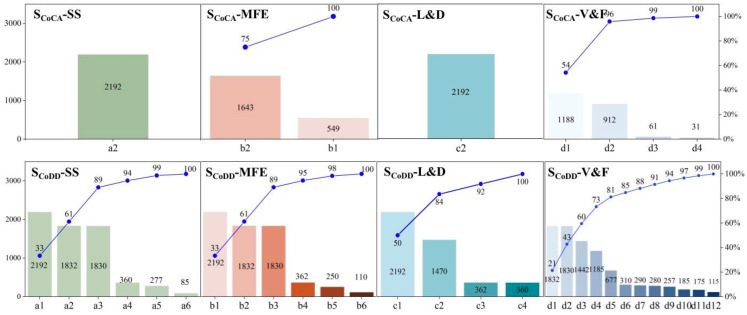
The frequency and cumulative frequencies of the various food items during the period from 2016 to 2021. The left axis represents the frequency, with a maximum frequency of 2192, which means that the food is part of the composition of the daily meal for the years 2016–2021, and the right axis represents the cumulative frequency.(Notes: al-potato; a2-standard flour; a3-buckwheat; a4-long-grain rice; a5-millet; a6-black rice; b1-egg; b2-bone-in pork; b3-grass carp; b4-carp; b5-chicken; b6-lean pork; c1-milk; c2-black beans; c3-red beans; c4-green beans; d1-carrot; d2-banana; d3-cabbage; d4-garlic sprout; d5-radish; d6-eggplant; d7-kidney beans; d8-silver ear fungus, white, dried; d9-apple; d10-leek; d11-rape cabbage; d12-wood ear fungus, dried).

Figure 4 shows the frequency and cumulative frequencies of the various food items during the period from 2016 to 2020 (a total of 2192 days). The O&F category is not discussed in this section, as it includes only soybean oil in all dietary scenarios. A total of nine foods are included in the S_CA_, all of which had the lowest cost per calorie among the food groups at any given time. Of these, SS and L&D include only one type of food each, namely, standard grade flour and black beans. There are differences in the degree of variation of the different foods in the V&F and MFE categories, leading to the occurrence of least-cost substitution. The highest to lowest frequencies in the V&F category are carrots, bananas, garlic moss, and cabbage, which means that carrots and bananas were the least expensive choices among the fruits and vegetables most of the time. The frequency of bone-in pork in the MFE category is almost three times higher than that of eggs. It can be seen that bone-in pork is the least-costly MFE option, and eggs are the least-costly alternative during the pork price increase (e.g., after February 2020).

Due to the need to consider food diversity, the S_BD_ includes a total of 28 food items, with 13 foods guaranteed to be part of the daily diet. In line with the “balanced cereal-based diet” proposed by the CDG, SS includes six food items in a descending order of frequency as follows: potato, standard flour, buckwheat, long-grain rice, millet, and black rice. As potatoes are the only food in the potato category for which the price data are collected, potatoes appear most frequently, while there are cost substitutes in refined cereals and whole cereals due to their rich composition. V&F includes 12 food items. The MFE category includes six food items, corresponding to the dietary guidelines for the consumption of meat, poultry, fish, and eggs. The frequency of eggs reaches a maximum, as only the price data for egg products were collected. Bone-in pork is the cheapest food on a calorific value basis in the MFE, so it is the most common meat in the least-cost diet, after eggs. However, to increase the MFE diversity, chicken and lean pork enter the S_BD_ as alternatives to bone-in pork, but this also results in meat costs that are 1–3 times higher. L&D includes milk and legumes, each with a frequency of 50%. For legumes, black beans, which have the lowest cost per calorie, are the most common, while red and green beans complete the variety. Compared to the S_CA_, the diversity costs of L&D in the S_BD_ are higher, increasing from approximately CNY 2.7 to CNY 3.2.

### 3.3. Dietary Nutrition Change

**Figure 5:**
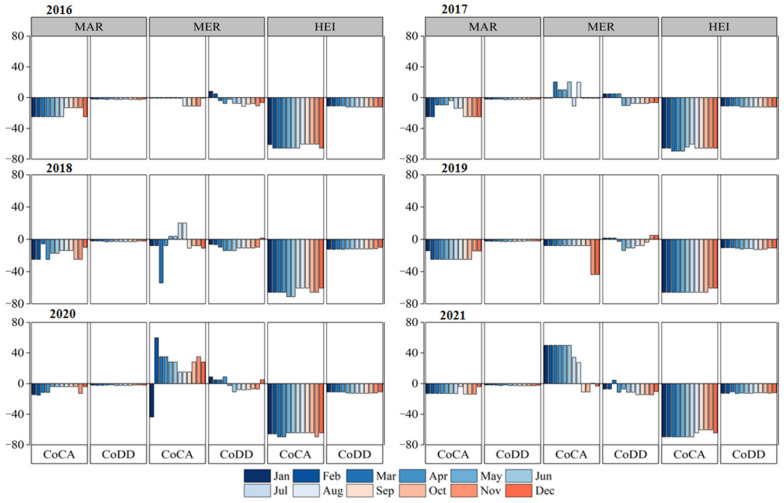
Deviation of dietary nutrient quality in CA and BD compared to the balanced diet pattern. If MAR, MER, and HEI levels are lower than those under a balanced diet, it shows a negative deviation, and a larger deviation indicates a lower nutritional level; conversely, a positive deviation indicates a higher nutritional level than the level under a balanced diet.

The effect of the changes in food prices on the nutrient content of the diet was analyzed by calculating the MAR, MER, and HEI, and Figure 5 shows the deviation of the MAR, MER, and HEI compared to the recommended diet by the CDG. Overall, the MAR and HEI of both the S_CA_ and S_BD_ have negative deviations, and the absolute value of the deviation of the S_CA_ is greater than that of the S_BD_, suggesting that a reduction in the dietary diversity would reduce the nutritional quality of the diet. The average deviation in the MAR during 2016–2021 for the S_CA_ is −16%, but it is significantly higher in 2020 and 2021 than in other periods, which is mainly due to the increase in pork prices since March 2020. Therefore, eggs were the cheapest animal food to substitute for pork, and the cost per unit of nutrient density of eggs over this period was only 20% of that of pork (Supplemental Table S5). Cabbage is also a low-cost food per unit of nutrient density, as shown in Figure 4, with significantly lower MAR deviations in the S_CA_ in March 2018, which is mainly due to replacing carrots with cabbage, which significantly increased protein, fiber, vitamin, and mineral availability, especially vitamin C, by almost five times. It should be noted, however, that according to the principle of equal calorie exchange, 260 g of eggs and 1470 g of cabbage per day would be required to replace 60 g pork and 720 g carrots, respectively, since the same quantity of eggs and cabbage contains 75% and 50% fewer calories, respectively, than pork and carrots. Although such a high consumption is unrealistic and not recommended, it does demonstrate the potential of eggs and cabbage to maintain dietary nutritional levels within price constraints. The mean deviation of the MAR for the S_BD_ was −2%, and the intakes of all beneficial nutrients, except fiber and Se, were close to or above the recommended intakes. The small difference in the MAR over time in the S_BD_ is mainly due to the restriction of dietary diversity, which makes the composition and nutrient content of the diet relatively stable, but also leads to a lack of flexible alternative foods and makes the cost of the diet more sensitive to fluctuations in food prices.

The MER of the S_CA_ and S_BD_ deviated very little from the balanced diet, with negative deviations that are mainly due to excessive sodium. The MER of the S_CA_ was better than those of the balanced diet, which was mainly due to the mono-consumption of MFE foods and, therefore, relatively low intakes of limiting nutrients such as fat and saturated fatty acids. The HEI of the S_CA_ was much lower than that of the balanced diet model, with a mean deviation of −65.2%, because only the cheapest foods from the five food groups were included in the S_CA_. In contrast, the S_BD_ had a wide variety of foods, so the deviation from the balanced diet was small (−12.0%). In the S_BD_, compared with the balanced diet, the food groups that did not meet the intake standard were vegetables, dark vegetables, and cereals (less than 90%), while among the food groups with restricted intakes, animal meat and edible oils exceeded the recommended upper limit by 12% and 6%, respectively.

Table 2 shows the contribution of each food group in terms of energy, macronutrients, and micronutrients under the two dietary scenarios. SS are the main source of energy, protein, carbohydrates, and thiamine, while V&F provide mainly fiber and vitamin C. L&D supply a majority of the required calcium and riboflavin. MFE provide most of the lipids and cholesterol, and O&F are the main source of lipids and vitamin E. The contribution of food groups to some nutrients varied between the scenarios; for example, the contribution of magnesium and iron from SS in the S_BD_ was almost 50%, while that from the S_CA_ was less than 25%, which was mainly due to differences in the whole grain intakes. The contribution of dietary fiber from L&D in the S_BD_ was approximately 1/3 that of the S_CA_, which was mainly due to differences in the legume intakes, while the calcium intake in the S_BD_ was twice that of the S_CA_, which was mainly due to added milk in the L&D group, whereas the S_CA_ included only legumes. These results emphasize the importance of dietary diversity in the satisfaction of nutritional needs.

## 4. Discussion

### 4.1. Cost Differences of Diet Patterns

Our main finding is that achieving a balanced dietary pattern based on the CDG leads to a significant increase in food expenditure, ranging from 20–120% for lowest-income and low-income groups, even for the cheapest food combinations. Based on the different income quintiles, we estimated that at least 45.71 million (the lowest 5% of urban residents) and 182.85 million urban households (the lowest 20% of urban residents) would need to increase their dietary expenditure to meet the basic energy requirements and healthy requirements, respectively. The premium for healthy requirements, as measured by the CoBD/CoCA ratio, varies between 1.68 and 1.95. This high premium is mainly because the S_BD_ follows the balanced diet requirement of “eating more than 12 food items in one day”, so different food items in the same food group should be included, rather than just the least-cost foods in the food group, as in the S_CA_. Compared to the lowest dietary cost of meeting daily energy needs in previous studies, the average in this study is CNY 7.63, or approximately USD 1.95 in 2011 purchasing power parity (PPP) terms, which is higher than the global average daily cost (USD 0.57 or USD 0.79) [2,10]. The main reason for this may be that in these studies, only the total energy intake was restricted, and the structure of the energy sources was not taken into account, which means that the most affordable starchy staples and fats were the main sources of energy; however, there are significant health risks in following this model. In contrast, in this study, the energy requirements were determined on the basis of the CDG-based balanced diet, that is, the shares of energy provided by SS, L&D, V&F, MFE, and O&F were 46.6%, 5.4%, 14.2%, 22.5%, and 11.3%, respectively, thus ensuring an adequate and balanced distribution of energy. The minimum cost of meeting the essential nutrient requirements in this study was USD 2.06 (in 2011 PPP), which is lower than the global average cost of meeting all essential nutritional requirements (USD 2.33) [2], the cost of meeting the EAT-Lancet reference diet (USD 2.84) [4] and the cost of meeting the UN definition of a healthy diet (USD 3.75) [2]. The main reason for this may be that a healthy diet encourages increased vegetable consumption and limited meat consumption; however, meat prices in Western countries are lower than those in China, and fruit and vegetable prices are higher than those in China, thus increasing food expenditure.

This study also found that the COVID-19 outbreak led to an increase in food costs, with the S_CA_ and S_BD_ costs being 13% and 12% higher per unit of energy supplied in February 2020 compared to the average for the study period, and the nutrient costs, mainly for fiber, thiamine, niacin, Fe, and Zn, increased by 26% and 13% for the S_CA_ and S_BD_, respectively, compared to the average. The data confirmed that the Chinese consumer price index (a measure of inflation) rose by 5.2% year-on-year in February, but the food prices soared by 21.9% [6], which was mainly due to higher pork and vegetable prices [36]. The nutritional status of vulnerable groups was affected by rising food prices and reduced incomes; e.g., groups of migrant workers who suffered income losses significantly reduced their nutritional intake as a result of disease prevention measures restricting people’s movement and preventing them from returning to work, and most of them had to reduce their food expenditure and buy large quantities of grains and other basic foods instead of nutritious foods [37].

Looking across food groups, our analysis confirms that fruits and vegetables are the most expensive components of the CDG-based diet, accounting for 43.4% (IQR 41.4–45.6%) of the cost of the S_CA_, compared to a smaller share of starchy staples and oils, with 17.1% (16.4–17.9%), which is in line with the results of the existing research (40% and 16%) [2]. For high-protein foods, the dietary diversity premium for L&D is 4.1, mainly because the S_CA_ covers only the cheapest legumes, while the S_BD_ covers not only legumes, but also the more expensive milk, and it costs three times as much to meet the guideline requirement of 300 ml of dairy per day. Staple starchy foods (buckwheat, black rice, and standard flour), pulses, and cabbage have the highest values in terms of nutrients per unit cost (Supplementary Table S5). Many of these foods offer a high nutrient density and low cost. However, foods with a high nutrient density are not always the most economical choice; for example, the high nutrient density of milk is offset by its high cost, so black beans offer a more affordable nutritional value.

### 4.2. Policy Implications

Nutrition is relevant to all 17 Sustainable Development Goals created by the UN [38]. In 2019, the FAO and the WHO proposed the concept of “Sustainable Healthy Diets” and agreed on related objectives [39]. These objectives are aimed at achieving optimal growth and development for all, as well as supporting physical and mental health and social well-being at all life stages for the contemporary and future generations. The importance and urgency of transitioning to sustainable healthy diets are widely recognized worldwide. “The State of Food Security and Nutrition in the World 2021” suggests that all countries need to adopt sustainable healthy diets by 2030 to minimize the costs of health and climate change [5].

Within a span of 40 years, China lifted approximately 750 million people out of absolute poverty and became the second-largest economy in the world [40]. By 2020, China completely eradicated absolute poverty, which means that they achieved the goal of providing enough calories for 1.4 billion people at an affordable cost. This is a major step forward toward the global goal of achieving zero hunger by 2030. However, the eradication of absolute poverty, as defined by the current poverty criteria, ensures only the affordability of basic calories, and further improvements in nutritional health need to be made [31]. The publication of the Chinese Dietary Guidelines and the National Nutrition Plan (2017–2030) represents important efforts by the government to improve the nutritional level of the population. These documents help people increase their food consistency and variety and provide scientific guidance on healthy eating, but if people cannot afford the cost of the balanced diets that these documents advocate for, it is difficult to implement national nutrition policies and programs, at least for low-income populations. To help policymakers understand the restrictive role of food cost in accessing a nutritious diet, a key descriptive contribution of this paper is that it reveals the characteristics and proportion of populations that cannot achieve a recommended diet due to high costs. Therefore, dietary costs should be considered when developing the CDG, and social protection programs should be reassessed in the light of more comprehensive dietary costs.

Relying on education and the promotion of nutritious foods is not enough to implement national nutrition programs and achieve the goal of a balanced diet, as research shows that nutrition education does not lead to the purchase of healthy foods by the poor, and only a reduction in the price of these foods will lead to a shift in consumption [2]. Globally, the cost of nutritious diets was incorporated into policy dashboards [41] and official reports [5] to promote healthy dietary choices for low-income groups and is a promising indicator for monitoring the changes in food systems in high-income countries [7], low-income countries [42], and globally [43], in response to shocks such as the COVID-19 pandemic [10]. In China, the main official monitoring data reflecting the cost of food are the Wholesale Price Index for total agricultural products and for various food groups (e.g., cereals, edible oil, vegetables, fruits, meats, and aquatic products), which are published daily by the Chinese Ministry of Agriculture and Rural Affairs. While these indexes can provide a comprehensive assessment and overall picture of the general level of wholesale agricultural prices and their changes, the disadvantage is that they do not truly reflect the impact of retail price changes on consumers’ food choices. China should therefore consider integrating the monitoring of the cost of nutritious diets into its existing price monitoring regime; monitoring changes in this indicator could show the extent to which policy and program interventions have improved the access to nutritious diets, focusing on the needs of low-income people seeking the most affordable food at any given time and place.

In addition to strengthening price monitoring, China needs to introduce more active agricultural policies to ensure nutritional security. Food security in China has long focused on the safety of starchy staples, especially rice, maize, and wheat, and policy guidance based on food prices tends to focus on individual food groups, with insufficient policy protection for the promotion of a nutritionally complete and balanced diet. The 2015 China Rural Work Conference formally put forward the term “Big Food Vision” [44], which stressed that while ensuring food supply, the effective supply of meats, vegetables, fruits, fish, seafood, and other types of food, should be ensured to meet people’s increasingly diverse food consumption needs. China also seems to need to rebalance its agricultural policies and incentives toward more nutrition-sensitive investments and policies at all levels of the food production and consumption chain to reduce the cost of dietary enrichment for consumers and increase the purchasing power and affordability of healthy diets for the most vulnerable. Moreover, with the shift from the “cereal perspective” to the “broad food perspective” and from “quantitative” to “qualitative” food adequacy, China should perhaps redraw the poverty line. After China eliminated absolute poverty in 2020, the problem of poverty shifted from absolute poverty to relative poverty, and accordingly, the new phase of drawing the poverty line should be more diverse in its needs, especially by taking into account the cost of nutritious meals, and not just energy sufficiency. It was found that from a nutritional health perspective, China’s poverty line should be raised to CNY 3159, which is higher than the current poverty line (CNY 2300 in 2011). Therefore, the existing poverty line needs to be adjusted upward if policy interventions plan to address the nutritional status of the poor [31].

### 4.3. Limitations

Potential limitations should be considered. First, due to the availability of the price data, the range of foods included in the study was not complete, and processed foods such as snacks and soft drinks were missing compared to existing studies. However, in view of the relatively high prices of most processed foods in China, there is a high probability that processed foods will not be included in the least-cost diet in the calculation process, so the uncertainty of the data limitation has less impact on the main results. Second, the least-cost diets in the study establish only a baseline, i.e., the minimum cost a person needs to spend to meet the dietary guidelines, and do not take into account food culture and preferences. The resulting diet may not be applicable to regions and populations with specific food cultures; in other words, the minimum cost of the diet may be underestimated. For example, the low price of pork has become a major component of MFE in low-cost diets, but Muslims do not eat pork and eat more beef and lamb, increasing the cost and, therefore, the number of people who cannot afford the diet. Therefore, future studies should include dietary preferences to achieve a more comprehensive assessment of affordability and more targeted policy recommendations.

## 5. Conclusions

This paper uses monthly retail food price data in urban China to simulate the two lowest-cost diet scenarios, S_CA_ and S_BD_, representing the lower and upper limits of the cost of following the Chinese dietary guidelines, respectively. This study finds that the S_CA_ and S_BD_ are more expensive than the actual food expenditure of the lowest-income and low-income households, with at least 45.71 million people spending less than the CoCA, and 182.85 million spending less than the CoBD. Fruits and vegetables accounted for the largest share of expenditure in both scenarios, followed by high-quality protein-rich foods such as pulses and dairy, while less than 16% was spent on cereals and potatoes. Regarding dietary composition, the S_BD_ includes a wider variety of foods to meet the dietary guidelines, increasing the nutrient intake by 17% relative to the S_CA_, but it requires an additional 81% increase in expenditure. Governments should focus on nutritious foods in food security and agricultural policies, include food price considerations in the formulation of dietary guidelines, and include nutritious foods in price monitoring and regulation. An increase in the poverty line will also aid in policy interventions on nutritional status and will help to achieve the goals of the “Healthy China 2030” initiative.

## Figures and Tables

**Table 1:** Recommended intakes for main food groups and subgroups in the Chinese Dietary Guidelines (CDG).

Food Group Names	Recommended Consumption Group	Food Subgroup Names	Recommended Consumption Subgroup
Starchy staples (SS)	250	Refined cereals	75
		Whole cereals and mixed beans	100
		Tubers and roots	75
Vegetables and fruits (V&F)	800	Vegetables	225
		Vegetables—DGLV or red/orange	225
		Fruits	300
Meat, fish, and eggs (MFE)	150	Meat	50
		Fish	50
		Eggs	50
Legumes and dairy (L&D)	325	Dairy products	300
		Legumes and nuts	25
Oils and fats (O&F)	25	Oils and fats	25

**Table 2:** Share of energy and partial nutrients in S_CA_ (S_BD_), by food group.

	S_CA_ (S_BD_), %				
	SS	MFE	F&V	L&D	O&F
Energy	40.1 (39.9)	16.6 (18.1)	18.3 (17.4)	13.6 (13.5)	11.4 (11.1)
Protein	43.4 (34.7)	17.7 (34.7)	8.3 (10.2)	30.5 (20.4)	0.0 (0.0)
Lipids	7.6 (7.9)	41.0 (38.0)	1.5 (1.8)	14.9 (17.8)	35.0 (34.5)
Carbohydrate	58.7 (60.9)	0.7 (0.6)	32.0 (29.7)	8.5 (8.8)	0.0 (0.0)
Dietary fiber	0.0 (14.7)	0.0 (0.0)	48.2 (66.5)	51.8 (18.8)	0.0 (0.0)
Elements					
Calcium	18.4 (16.0)	12.7 (9.2)	27.3 (25.4)	40.8 (49.1)	0.9 (0.2)
Phosphorus	36.8 (36.5)	14.1 (21.9)	15.1 (13.1)	33.8 (28.4)	0.2 (0.1)
Magnesium	24.6 (47.9)	3.2 (7.5)	35.3 (29.8)	36.7 (14.7)	0.2 (0.1)
Iron	10.7 (47.5)	13.5 (10.7)	33.2 (27.9)	38.4 (11.5)	4.1 (2.4)
Zinc	7.3 (37.2)	21.9 (23.5)	19.7 (20.4)	46.5 (16.0)	4.5 (3.0)
Selenium	43.2 (26.7)	33.4 (55.7)	11.2 (13.7)	12.1 (4.0)	0.0 (0.0)
Vitamins					
Thiamin	67.6 (60.6)	12.7 (14.7)	10.7 (15.2)	9.0 (9.5)	0.0 (0.0)
Riboflavin	15.2 (18.1)	27.4 (22.3)	26.8 (22.9)	30.7 (36.8)	0.0 (0.0)
Niacin	37.5 (43.0)	19.5 (33.2)	30.9 (21.2)	12.0 (2.6)	0.0 (0.0)
Folate	11.9 (19.4)	24.7 (18.7)	30.6 (46.7)	32.8 (15.2)	0.0 (0.0)
Vitamin C	0.0 (10.4)	0.0 (0.0)	100.0 (71.3)	0.0 (0.0)	0.0 (0.0)
Vitamin E	1.9 (18.0)	0.1 (6.0)	3.0 (6.0)	31.7 (9.3)	63.4 (59.0)
Limited-intake nutrients					
SFA	0.0 (0.4)	100.0 (64.8)	0.0 (0.0)	0.0 (34.8)	0.0 (0.0)
Cholesterol	0.0 (0.0)	100.0 (88.7)	0.0 (0.0)	0.0 (11.3)	0.0 (0.0)
Na	2.1 (1.4)	41.8 (21.1)	55.2 (28.6)	0.6 (48.9)	0.4 (0.0)

## Data Availability

The data presented in this study are available upon request from the corresponding authors.

## Supplementary Materials

The following supporting information can be downloaded at https://www.mdpi.com/article/10.3390/nu15122704/s1, Table S1: Food Items, Food Groups, and Food Composition; Table S2: Average monthly price in 1000 kcal of various food items (CNY); Table S3: Healthy Eating Index (HEI) components for Chinese residents (EER = 2000 kcal); Table S4: Nutritional constraints implemented in MAR and MER; Table S5: Cost of beneficial nutrients for various food groups in S_CA_ and S_BD_.

## References

[1] FAO Rome (1996). Declaration on World Food Security and World Food Summit Plan of Action.

[2] Herforth A., Bai Y., Venkat A., Mahrt K., Ebel A., Masters W.A. (2020). Cost and Affordability of Healthy Diets across and within Countries; Background Paper for the State of Food Security and Nutrition in the World 2020.

[3] Raghunathan K., Headey D., Herforth A. (2021). Affordability of Nutritious Diets in Rural India. Food Policy.

[4] Hirvonen K., Bai Y., Headey D., Masters W.A. (2020). Affordability of the EAT–Lancet Reference Diet: A Global Analysis. Lancet Glob. Health.

[5] FAO, IFAD, UNICEF, WFP, WHO (2020). The State of Food Security and Nutrition in the World 2020-Transforming Food Systems for Affordable Healthy Diets.

[6] Fan S., Si W., Zhang Y. (2020). How to Prevent a Global Food and Nutrition Security Crisis under COVID-19?. China Agric. Econ. Rev..

[7] Akter S. (2020). The Impact of COVID-19 Related ‘Stay-at-Home’ Restrictions on Food Prices in Europe: Findings from a Preliminary Analysis. Food Sec..

[8] Headey D., Ruel M. The COVID-19 Nutrition Crisis: What to Expect and How to Protect, IFPRI 2020. https://www.ifpri.org/blog/covid-19-nutrition-crisis-what-expect-and-how-protect.

[9] O’Connell M., Smith K., Stroud R. (2022). The Dietary Impact of the COVID-19 Pandemic. J. Health Econ..

[10] Bai Y., Alemu R., Block S.A., Headey D., Masters W.A. (2021). Cost and Affordability of Nutritious Diets at Retail Prices: Evidence from 177 Countries. Food Policy.

[11] Headey D.D., Alderman H.H. (2019). The Relative Caloric Prices of Healthy and Unhealthy Foods Differ Systematically across Income Levels and Continents. J. Nutr..

[12] Masters W.A., Bai Y., Herforth A., Sarpong D.B., Mishili F., Kinabo J., Coates J.C. (2018). Measuring the Affordability of Nutritious Diets in Africa: Price Indexes for Diet Diversity and the Cost of Nutrient Adequacy. Am. J. Agric. Econ..

[13] Chastre C., Duffield A., Kindness H., LeJeune S., Taylor A. (2007). The Minimum Cost of a Healthy Diet.

[14] Beydoun M.A., Powell L.M., Chen X., Wang Y. (2011). Food Prices Are Associated with Dietary Quality, Fast Food Consumption, and Body Mass Index among U. S. Children and Adolescents. J. Nutr..

[15] Dizon F., Herforth A., Wang Z. (2019). The Cost of a Nutritious Diet in Afghanistan, Bangladesh, Pakistan, and Sri Lanka. Glob. Food Secur..

[16] Miller V., Yusuf S., Chow C.K., Dehghan M., Corsi D.J., Lock K., Popkin B., Rangarajan S., Khatib R., Lear S.A. (2016). Availability, Affordability, and Consumption of Fruits and Vegetables in 18 Countries across Income Levels: Findings from the Prospective Urban Rural Epidemiology (PURE) Study. Lancet Glob. Health.

[17] Clements K.W., Si J. (2018). Engel’s Law, Diet Diversity, and the Quality of Food Consumption. Am. J. Agric. Econ..

[18] Harris J., Tan W., Raneri J.E., Schreinemachers P., Herforth A. (2022). Vegetables for Healthy Diets in Low- and Middle-Income Countries: A Scoping Review of the Food Systems Literature. Food Nutr. Bull..

[19] Dolislager M., Liverpool-Tasie L.S.O., Mason N.M., Reardon T., Tschirley D. (2022). Consumption of Healthy and Unhealthy Foods by the African Poor: Evidence from Nigeria, Tanzania, and Uganda. Agric. Econ..

[20] Schneider K.R., Christiaensen L., Webb P., Masters W.A. (2022). Assessing the Affordability of Nutrient-adequate Diets. Am. J. Agric. Econ..

[21] Yin J., Zhang X., Huang W., Liu L., Zhang Y., Yang D., Hao Y., Chen Y. (2021). The Potential Benefits of Dietary Shift in China: Synergies among Acceptability, Health, and Environmental Sustainability. Sci. Total Environ..

[22] Huang L., Wang Z., Wang H., Zhao L., Jiang H., Zhang B., Ding G. (2021). Nutrition Transition and Related Health Challenges over Decades in China. Eur. J. Clin. Nutr..

[23] Chinese Nutrition Society (2022). The Chinese Dietary Guidelines, 2022 ed..

[24] National Bureau of Statistics (2013). China Urban Life and Price Yearbook.

[25] Yang Y. (2019). Chinese Food Composition Tables (Standard Edition) (Book 1).

[26] Yang Y. (2019). Chinese Food Composition Tables (Standard Edition) (Book 2).

[27] Stigler G.J. (1945). The Cost of Subsistence. J. Farm Econ..

[28] Bai J., Wang L., Wang H., Wang Z., Zhang B. (2022). Intakes of energy and macronutrient from Chinese 15 provinces (autonomous regions and municipalities) adults aged 18 to 35 in 1989–2018. J. Hyg. Res..

[29] (2022). Food Prices for Nutrition Technical Assistance Tools for Calculating the Cost of a Healthy Diet, Version 3.0. https://sites.tufts.edu/foodpricesfornutrition/tools/.

[30] Ambikapathi R., Schneider K.R., Davis B., Herrero M., Winters P., Fanzo J.C. (2022). Global Food Systems Transitions Have Enabled Affordable Diets but Had Less Favourable Outcomes for Nutrition, Environmental Health, Inclusion and Equity. Nat. Food.

[31] Li L., Bai J., Zhang C. (2020). Impacts of Income on the Dietary Health of Chinese Rural Residents from the Perspective of Poverty-Line: Based on the China Nutrition and Health Survey Data. Res. Agric. Mod..

[32] Yuan Y., Li F., Dong R., Chen J., He G., Li S., Chen B. (2017). The Development of a Chinese Healthy Eating Index and Its Application in the General Population. Nutrients.

[33] Vieux F., Soler L., Touazi D., Darmon N. (2013). High Nutritional Quality Is Not Associated with Low Greenhouse Gas Emissions in Self-Selected Diets of French Adults. Am. J. Clin. Nutr..

[34] Perignon M., Sinfort C., El Ati J., Traissac P., Drogué S., Darmon N., Amiot M.J., Achir N., Alouane L. (2019). How to Meet Nutritional Recommendations and Reduce Diet Environmental Impact in the Mediterranean Region?. An Optimization Study to Identify More Sustainable Diets in Tunisia. Glob. Food Secur..

[35] Chinese Nutrition Society (2014). Chinese Dietary Nutrients Reference Intakes, 2013 ed..

[36] Yu X., Liu C., Wang H., Feil J. (2020). The Impact of COVID-19 on Food Prices in China: Evidence of Four Major Food Products from Beijing, Shandong and Hubei Provinces. China Agric. Econ. Rev..

[37] Rozelle S., Rahimi H., Wang H., Dill E. (2020). Lockdowns Are Protecting China’s Rural Families From COVID-19, But the Economic Burden Is Heavy. COVID-19 and Global Food Security.

[38] Grosso G., Mateo A., Rangelov N., Buzeti T., Birt C. (2020). Nutrition in the Context of the Sustainable Development Goals. Eur. J. Public Health.

[39] FAO, WHO (2019). Sustainable Healthy Diets—Guiding Principles.

[40] Biswas A.K., Tortajada C. How China Eradicated Absolute Poverty? China Daily, 12 April 2021. https://www.chinadailyhk.com/article/a/162843#:~:text=Monday%2C%20April%2012%2C%202021%2C%2012%3A59%20By%20Asit%20K.,Brazil%20%28US%241%2C947.28%29%20and%2073%20percent%20of%20India%20%28US%24266.58%29..

[41] Fanzo J., Haddad L., McLaren R., Marshall Q., Davis C., Herforth A., Jones A., Beal T., Tschirley D., Bellows A. (2020). The Food Systems Dashboard Is a New Tool to Inform Better Food Policy. Nat. Food.

[42] Narayanan S., Saha S. (2020). Urban Food Markets and the Lockdown in India.

[43] Masters W.A. (2020). COVID-19 Disruptions and Resilience of Retail Food Prices Around the World. https://poverty-action.org/recovr-study/covid-19-disruptions-and-resilience-retail-food-prices-around-world.

[44] The China Rural Work Conference. http://www.moa.gov.cn/ztzl/ncgzhy2015/zxdt/201512/t20151226_4966987.htm.

