# Supplementary material for: Healthy Eating for All? The Challenge of Adhering to Dietary Guidelines for Low-Income Groups in China
Source: Nutrients. 2023 Jun 9;15(12):2704. doi: 10.3390/nu15122704 (PMC10301239; doi:10.3390/nu15122704)
Supplement: Supplementary file 1 [file nutrients-15-02704-s001.zip › nutrients-2416961-supplementary.pdf]

Supplementary Information for

Healthy Eating for All? The Challenge of Adhering to Dietary Guidelines for Low-Income Groups in China

The monitoring of prices is centered around the large agricultural product wholesale and retail markets in 36 large- and medium-sized cities, including the four municipalities of Beijing, Shanghai, Tianjin, and Chongqing; 27 provincial capitals such as Guangzhou, Wuhan, Harbin, Shenyang, Chengdu, Nanjing, Xi'an, Changchun, Jinan, Hangzhou, Taiyuan, Changsha, Urumqi, Zhengzhou, Kunming, Lanzhou, Guiyang, Hefei, Shijiazhuang, Fuzhou, Nanning, Hohhot, Nanchang, Haikou, Xining, Yinchuan, and Lhasa; and five planned single-city districts, which are Dalian, Qingdao, Shenzhen, Xiamen, and Ningbo.

Table S1. Food Items, Food Groups, and Food Composition

Items	Group	Edible portion	Cal	Pro	Fat	CHO	Dietary	SFA	Thia-min	Ribofl-avin	Niacin	Folic acid	Vita-min C	Ca	P	K	Na	Mg	Fe	Zn	Se
			/100g edible portion																		
			kcal	g	g	g	fiber g	g	mg	mg	mg	ug	mg	mg	mg	mg	mg	mg	mg	mg	mg
Long-grain rice	SS	1.00	346	7.9	0.9	77.2	0.6	0.1	0.15	0.04	2.00	23.7	0	8	112	112	1.8	31	1.1	1.54	2.83
Polished round-grained rice	SS	1.00	335	7.3	0.4	75.7	0.4	0.3	0.08	0.04	1.10	6.8	0	24	80	58	6.2	25	0.9	1.07	2.49
Special flour	SS	1.00	351	10.3	1.1	75.2	0.6	0.2	0.17	0.06	2.00	47.4	0	27	114	128	2.7	32	2.7	0.97	6.88
Standard flour	SS	1.00	362	15.7	2.5	70.9	0.0	0.5	0.46	0.05	1.91	20.7	0	31	167	190	3.1	50	0.6	0.20	7.42
Potato	SS	0.75	81	2.6	0.2	17.8	1.1	0.0	0.10	0.02	1.10	15.7	14	7	46	347	5.9	24	0.4	0.30	0.47
Corn flour	SS	1.00	352	8.0	4.5	73.1	6.2	0.6	0.34	0.06	3.00	45.1	0	12	187	276	0.5	111	1.3	1.22	1.58

Items	Group	Edible portion	/100g edible portion																		
			Cal	Pro	Fat	CHO	Dietary	SFA	Thia-min	Ribofl-avin	Niacin	Folic acid	Vita-min C	Ca	P	K	Na	Mg	Fe	Zn	Se
			kcal	g	g	g	fiber g	g	mg	mg	mg	ug	mg	mg	mg	mg	mg	mg	mg	mg	mg
Millet	SS	1.00	361	9.0	3.1	75.1	1.6	2.5	0.33	0.10	1.50	29.6	0	41	229	284	4.3	107	5.1	1.87	4.74
Black rice	SS	1.00	341	9.4	2.5	72.2	3.9	0.7	0.33	0.13	7.90	--	0	12	356	256	7.1	147	1.6	3.80	3.20
Glutinous rice	SS	1.00	345	9.0	1.0	75.3	0.6	--	0.10	0.03	2.30	39.6	0	26	113	137	1.5	49	1.4	1.54	2.71
Buckwheat	SS	1.00	340	11.3	2.8	70.2	0.0	0.5	0.26	0.10	3.47	29.1	0	71	243	304	0.9	151	7.0	1.94	2.16
Red bean	L&D	1.00	324	20.2	0.6	63.4	7.7	0.1	0.16	0.11	2.00	151.9	--	74	305	860	2.2	138	7.4	2.20	3.80
Green bean	L&D	1.00	329	21.6	0.8	62.0	6.4	0.2	0.25	0.11	2.00	286.2	0	81	337	787	2.2	138	7.4	2.20	3.80
Black bean	L&D	1.00	401	36.0	15.9	33.6	10.2	2.3	0.20	0.33	2.00	186.4	0	224	500	1377	3.0	243	7.0	4.18	6.79
Milk	L&D	1.00	67	3.4	3.7	5.1	0.0	2.1	0.02	0.12	0.00	3.5	0	113	103	127	120.3	12	0.3	0.24	0.00
Soybean oil	O&F	1.00	899	Tr	99.9	0.0	--	15.0	Tr	Tr	Tr	Tr	0	13	7	3	4.9	3	2.0	1.09	0.00
Groundnut oil	O&F	1.00	899	Tr	99.9	0.0	--	18.4	Tr	Tr	Tr	Tr	--	12	15	1	3.5	2	2.9	0.48	--
Blend oil	O&F	1.00	900	Tr	99.9	0.1	--	14.2	Tr	0.09	0.10	Tr	0	75	0	2	10.5	24	4.1	1.27	0.00
Rapeseed oil	O&F	1.00	899	Tr	99.9	0.0	--	7.0	Tr	Tr	Tr	Tr	--	9	9	2	7.0	3	3.7	0.54	--
Egg	MFE	0.88	139	13.1	8.6	2.4	0.0	4.6	0.09	0.20	0.20	113.3	Tr	56	130	154	131.5	10	1.6	0.89	13.96
Chicken	MFE	0.52	145	20.3	6.7	0.9	0.0	0.8	0.06	0.07	7.54	6.5	Tr	13	166	249	62.8	22	1.8	1.46	11.92
Bone-in pork	MFE	0.96	568	9.3	59.0	0.0	0.0	26.7	0.31	0.10	5.30	10.9	Tr	6	96	214	80.0	17	1.0	1.61	3.70
Lean pork	MFE	1.00	153	20.7	7.8	0.0	0.0	3.0	0.31	0.10	5.30	8.1	Tr	2	141	305	54.0	23	1.1	2.20	8.32

Items	Group	Edible portion	/100g edible portion																			
			Cal	Pro	Fat	CHO	Dietary	SFA	Thia-min	Ribofl-avin	Niacin	Folic acid	Vita-min C	Ca	P	K	Na	Mg	Fe	Zn	Se
			kcal	g	g	g	fiber g	g	mg	mg	mg	ug	mg	mg	mg	mg	mg	mg	mg	mg	mg
Bone-in mutton	MFE	0.79	110	19.5	3.4	0.3	0.0	1.5	0.05	0.19	6.00	3.7	Tr	6	182	143	60.0	20	2.7	2.18	4.49
Beef	MFE	1.00	160	20.0	8.7	0.5	0.0	4.1	0.04	0.11	4.15	3.6	Tr	5	182	212	64.1	22	1.8	4.70	3.15
Mutton, without bone	MFE	1.00	139	18.5	6.5	1.6	0.0	4.2	0.07	0.16	4.41	3.7	Tr	16	161	300	89.9	23	3.9	3.52	5.95
Grass carp	MFE	0.58	113	16.6	5.2	0.0	0.0	1.0	0.04	0.11	2.80	19.8	Tr	38	203	312	46.0	31	0.8	0.87	6.66
Carp	MFE	0.54	109	17.6	4.1	0.5	0.0	0.8	0.03	0.09	2.70	36.4	Tr	50	204	334	53.7	33	1.0	2.08	15.38
Cabbage	F&V	0.89	20	1.6	0.2	3.4	0.9	--	0.05	0.04	0.65	18.5	38	57	33	134	68.9	12	0.8	0.46	0.57
Radish	F&V	0.95	16	0.7	0.1	4.0	0.0	--	0.02	0.01	0.14	27.0	19	47	16	167	54.3	12	0.2	0.14	0.12
Cucumber	F&V	0.92	16	0.8	0.2	2.9	0.5	--	0.02	0.03	0.20	9.1	9	24	24	102	4.9	15	0.5	0.18	0.38
Eggplant	F&V	0.93	23	1.1	0.2	4.9	1.3	--	0.02	0.04	0.60	12.2	5	24	23	142	5.4	13	0.5	0.23	0.48
Green pepper	F&V	0.91	22	0.8	0.3	5.2	--	--	0.02	0.02	0.62	69.4	59	11	20	154	7.0	15	0.3	0.21	0.02
Silver ear fungus, white, dried	F&V	0.96	261	10.0	1.4	67.3	30.4	0.5	0.05	0.25	5.30	--	0	36	369	1588	82.1	54	4.1	3.03	2.95
Wood ear fungus, dried	F&V	0.96	265	12.1	1.5	65.6	29.9	0.3	0.17	0.44	2.50	81.6	--	247	292	757	48.5	152	97.4	3.18	3.72

Items	Group	Edible portion	/100g edible portion																		
			Cal	Pro	Fat	CHO	Dietary	SFA	Thia-min	Ribofl-avin	Niacin	Folic acid	Vita-min C	Ca	P	K	Na	Mg	Fe	Zn	Se
			kcal	g	g	g	fiber g	g	mg	mg	mg	ug	mg	mg	mg	mg	mg	mg	mg	mg	mg
Kidney bean	F&V	0.96	35	2.2	0.2	7.4	2.6	--	0.06	0.04	0.90	--	39	26	40	192	9.5	28	0.8	0.60	1.60
Ginger	F&V	0.95	46	1.3	0.6	10.3	2.7	--	0.02	0.03	0.80	3.5	4	27	25	295	14.9	44	1.4	0.34	0.56
Garlic sprout	F&V	0.90	66	2.0	0.1	15.4	2.5	--	0.04	0.07	0.20	90.9	1	19	52	161	3.8	28	4.2	1.04	2.17
Garlic	F&V	0.85	12	4.5	0.2	27.6	1.1	--	0.04	0.06	0.60	--	7	39	117	302	19.6	21	1.2	0.88	3.09
Carrot	F&V	0.89	39	1.0	0.2	8.8	1.1	--	0.04	0.03	0.60	20.4	13	32	27	190	71.4	14	1.0	0.23	0.63
Rape cabbage	F&V	0.96	19	1.8	0.2	2.9	0.9	--	0.01	0.10	0.00	107.6	24	191	34	143	98.8	34	5.9	1.27	0.00
Tomato	F&V	0.91	15	0.9	0.2	3.3	′-	--	0.02	0.01	0.49	8.3	14	4	24	179	9.7	12	0.2	0.12	Tr
Celery	F&V	0.85	17	0.6	0.1	4.8	2.2	--	0.01	0.03	0.22	13.6	4	36	35	15	313.3	15	0.2	0.10	0.10
Leek	F&V	0.90	25	2.4	0.4	4.5	0.0	--	0.04	0.05	0.86	61.2	2	44	45	241	5.8	24	0.7	0.25	1.33
Water-melon	F&V	0.59	31	0.5	0.3	6.8	0.2	--	0.02	0.04	0.30	4.0	6	7	12	97	3.3	14	0.4	0.09	0.09
Banana	F&V	0.59	93	1.4	0.2	22.0	1.2	--	0.02	0.04	0.70	20.2	8	7	28	256	0.8	43	0.4	0.18	0.87
Apple	F&V	0.85	53	0.4	0.2	13.7	1.7	--	0.02	0.02	0.20	6.3	3	4	7	83	1.3	4	0.3	0.04	0.10

Notes: Data from Chinese Food Composition Tables [1-2]. Special symbols in the table are described as follows. “--”：theoretically, a certain amount of this ingredient should be present in the food, but in reality, it was not found to be present. “Tr”：not detected at all, or detected as a trace.

Table S2. Average monthly price in 1000 kcal of various food items (CNY)

Food Group	Items	Average Price	Food Group	Items	Average Price
Vegetables and Fruit (V&F)	Wood ear fungus, dried	36.13	Starchy staples (SS)	Long-grain rice	1.51
	Cabbage	17.66		Polished round-grained rice	1.55
	Cucumber	44.08		Potato	6.73
	Eggplant	31.45		Special flour	1.49
	Garlic	10.80		Standard flour	1.34
	Garlic sprout	19.35		Black rice	3.33
	Ginger	25.74		Buckwheat	2.46
	Green pepper	42.15		Corn flour	1.74
	Kidney bean	33.01		Glutinous rice	2.36
	Radish	20.94		Millet	3.10
	Silver ear fungus, white, dried	33.61	Meat, fish and eggs (MFE)	Beef, steak, sirloin	38.62
	Carrot	13.12		Chicken	19.94
	Celery	46.84		Mutton, without bone	58.83
	Leek	34.58		Bone-in mutton	72.32
	Rape cabbage	33.51		Bone-in pork	5.75
	Tomato	51.76		Lean pork	22.25
	Apple	24.69		Carp	28.12
	Banana	12.41		Grass carp	13.80
	Watermelon	28.16		Egg	7.48

Legumes and Dairy (L&D)	Black bean	3.16	Oils and fats(O&F)	Blend oil	0.64
	Green bean	3.63		Groundnut oil	1.47
	Red bean	3.98		Rapeseed oil	0.70
	Milk	17.72		Soybean oil	0.54

Table S3. Healthy Eating Index (HEI) components for Chinese residents (EER = 2000 kcal)

Category	Food Groups	RI（g/d）	SP[1]	Weights	Score
Food Group	Total Grains	250	5	5	0 → 5
	--Whole Grains	50-100	2	10	0 → 10
	Tubes	75	0.8	5	0 → 5
	Vegetables	450	4.5	10	0 → 10
	--Dark Vegetables	225	2.25	5	0 → 5
	Fruits	300	3	10	0 → 10
	Dairy	300	1.2	10	0 → 10
	Legumes	15	0.8	5	0 → 5
	Seeds and Nuts	10	1	5	0 → 5
	Fish and Seafood	50	1.1	5	0 → 5
	Eggs	50	1.1	5	0 → 5
	Poultry	50	1.1	5	0 → 5
Restricted Food Group	Red Meat	25	0.8	10	0 → 10
	Cooking Oils	25	25	10	0 → 10

Notes: [1] SP is an abbreviation for standard portion, which is a food standard that is substituted according to the principle of consistency of energy, protein, fat, and carbohydrates. The SP values in this study refer to Yuan's research. A description of the way in which the weights are set is as follows. Most foods in this study were given a weight of 5, but whole grains, vegetables, fruit, dairy, red meat, and cooking oil were given a weight of 10 for the following main reasons: (1) Whole grains are an important source of dietary fiber and B vitamins, and increasing their intake is associated with a reduced risk of type 2 diabetes, cardiovascular disease, and cancer. (2) Fruits and vegetables are rich in vitamins, minerals, and dietary fiber, and are low in energy, which are important for satisfying the body's micronutrient needs, maintaining normal intestinal function, and reducing the risk of cardiovascular disease and lung cancer. (3) Dairy products are an important source of dietary calcium and high-quality protein, and increasing dairy intake has a certain effect on increasing bone density in children. (4) Surveys have shown that the intake of vegetables in China is declining, and the intake of fruit, milk, and whole grains is chronically insufficient, which is an important constraint to a balanced diet and leads to the insufficient intake of certain micronutrients [3]; therefore, the weight of these four food groups was increased to 10. (5) The excessive consumption of cooking oil is a long-standing feature of the Chinese diet, and this can increase fat intake, leading to a higher than optimal dietary fat-to-energy ratio and an increased risk of cardiovascular disease from the excessive

consumption of trans fatty acids. (6) As the main meat component of diets, the excessive consumption of livestock meat, which is high in saturated fatty acids, may increase the risk of obesity and cardiovascular disease, so both cooking oil and livestock meat were assigned a weight of 10 [4].

Table S4. Nutritional constraints implemented in MAR and MER

Nutritional Constraint Type	RNI
Total energy (kcal)	2000
Proteins (%E)	15-20
Fats (%E)	20-30
Carbohydrates (%E)	50-75
Fibers (g/d)	≥25
Saturated fatty acid (%E)	＜10
Thiamin (mg/d)	≥1.4/1.2 (male/female)
Riboflavin (mg/d)	≥1.4/1.2 (male/female)
Niacin (mg/d)	≥12-15
Folic acid (ug/d)	≥400
Vitamin C (mg/d)	≥100
Ca (mg/d)	≥800
P (mg/d)	≥720
K (mg/d)	≥2000
Na (mg/d)	＜2759/2365 (male/female)
Mg (mg/d)	≥330
Fe (mg/d)	≥12/20 (male/female)
Zn (mg/d)	≥12.5/7.5 (male/female)
Se (mg/d)	≥60

Notes: The RNI of proteins and Na refer to M. Perignon's paper [5], and total fats, SFA, fibers, vitamins, and minerals (except for Na) refer to the recommendations for those who are 18 years of age and older in the China Food Composition Tables [2].

Table S5. Cost of beneficial nutrients for various food groups in S_{CA} and S_{BD}

Item	Group	MAR/Cost	Item	Group	MAR/Cost
Potato	SS	13.42	Banana	V&F	6.70
Long-grain rice	SS	14.10	Garlic sprout	V&F	9.74
Buckwheat	SS	20.45	Cabbage	V&F	**24.36**
Black rice	SS	17.92	Carrots	V&F	9.67

Millet	SS	13.42	Apple	V&F	1.57
Standard flower	SS	**21.23**	Watermelon	V&F	5.45
Grass carp	MFE	9.95	White radish	V&F	12.26
Carp	MFE	6.63	Eggplant	V&F	5.33
Chicken	MFE	5.40	Kidney beans	V&F	9.02
Lean pork	MFE	4.19	Silver ear fungus, white, dried	V&F	3.36
Eggs	MFE	**10.92**	White radish	V&F	11.11
Bone-in pork	MFE	3.32	Cucumber	V&F	5.86
Black beans	L&D	**28.40**	Green pepper	V&F	10.13
Green beans	L&D	21.61	Wood ear fungus, dried	V&F	7.67
Red beans	L&D	19.95	Leek	V&F	7.37
Local milk (fresh)	L&D	3.54	Rape cabbage	V&F	17.61
Soybean oil	O&F	3.55			

References

1. Yang, Y. *Chinese Food Composition Tables (Standard Edition)(Book 1)*; Peking University Medical Press: Beijing, China, 2019; pp.27-226.
2. Yang, Y. *Chinese Food Composition Tables (Standard Edition)(Book 2)*; Peking University Medical Press.: Beijing, China, 2019; pp.51-333.
3. Chinese Nutrition Society. *The Chinese Dietary Guidelines (2022 Edition).* People's medical publishing house: Beijing, China, 2022; pp.93-304.
4. Yuan, Y.; Li, F.; Dong, R.; Chen, J.; He, G.; Li, S.; Chen, B. The Development of a Chinese Healthy Eating Index and Its Application in the General Population. *Nutrients* **2017**, *9*, 977, doi:10.3390/nu9090977.
5. Perignon, M.; Sinfort, C.; El Ati, J.; Traissac, P.; Drogué, S.; Darmon, N.; Amiot, M.J.; Achir, N.; Alouane, L.; et al. How to Meet Nutritional Recommendations and Reduce Diet Environmental Impact in the Mediterranean Region? An Optimization Study to Identify More Sustainable Diets in Tunisia. *Global Food Security* **2019**, *23*, 227–235, doi:10.1016/j.gfs.2019.07.006.
